# Peritoneal Lavage Cytology and Radiologically Evident Peritoneal Carcinomatosis Within 12 Months in Advanced Digestive Cancer Without Macroscopic Peritoneal Disease: A Single-Centre Prognostic Study

**DOI:** 10.3390/jcm15197485

**Published:** 2026-09-26

**Authors:** Horea-Florin Bocșe, Andra Ciocan, Roxana Popa, Bobe Petrushev, Nadim Al Hajjar, Florin Vasile Zaharie

**Affiliations:** 1 Department of Surgery, Faculty of Medicine, “Iuliu Hațieganu” University of Medicine and Pharmacy, Victor Babeș Street, No. 8, 400012 Cluj-Napoca, Romania; 2 Department of Surgery, “Octavian Fodor” Regional Institute of Gastroenterology and Hepatology, Croitorilor Street, No. 19–21, 400162 Cluj-Napoca, Romania; 3 Department of Pathology, “Octavian Fodor” Regional Institute of Gastroenterology and Hepatology, Croitorilor Street, No. 19–21, 400162 Cluj-Napoca, Romania

**Keywords:** peritoneal lavage, peritoneal cytology, CY1, occult peritoneal dissemination, digestive cancer, cell block, surgical staging, staging laparoscopy, free tumour cells, precision surgery

## Abstract

**Background/Objectives:** Peritoneal dissemination is a major pattern of failure in advanced digestive cancer and is poorly detected by cross-sectional imaging. Outside gastric cancer, peritoneal lavage cytology is rarely performed and carries no staging weight. We examined the association between cytological status and subsequent peritoneal carcinomatosis in patients without peritoneal disease on imaging or at operation. **Methods:** Retrospective prognostic study of consecutive patients with clinically staged T3–T4 digestive malignancy operated on by one surgical team (January 2019–December 2022). Lavage was performed before tumour manipulation and processed as cell blocks. The exposure was cytological status, positive (CY1) or negative (CY0); the outcome was radiologically evident peritoneal carcinomatosis within 12 months on computed tomography at 3, 6, 9 and 12 months, analysed as interval-censored data. **Results:** Of 62 patients, 10 (16.1%) had carcinomatosis at operation despite negative imaging. Of the 45 with digestive primaries and no peritoneal disease, 13 (28.9%) were CY1. Carcinomatosis developed in 10 of 13 CY1 and 5 of 32 CY0 patients (76.9% versus 15.6%; relative risk 4.92, 95% CI 2.09–11.62), an association preserved in patients without distant metastases and in those who underwent resection. The hazard ratio from a discrete-time proportional hazards model was 9.07 (95% CI 3.15–29.72; *p* = 0.00005). No clinicopathological variable examined was significantly associated with cytological status. **Conclusions:** In this exploratory series, positive lavage cytology was associated with early radiologically evident peritoneal carcinomatosis. The association is unadjusted and derives from a small, heterogeneous single-centre cohort; it does not establish independent prognostic value, nor does it support routine lavage cytology or changes to postoperative surveillance. It warrants prospective evaluation.

## 1. Introduction

Peritoneal dissemination is among the most consequential patterns of failure in advanced digestive malignancy. Roughly a quarter of patients with locally advanced or perforated colon cancer are at risk of developing peritoneal metastases, frequently without curative options once established [1,2]. Risk factors for metachronous peritoneal disease have been characterised in systematic reviews [3,4], yet prognosis in overt carcinomatosis remains poor despite three decades of refinement in cytoreductive surgery and intraperitoneal therapy [5].

The clinical difficulty is compounded by a diagnostic one. Cross-sectional imaging performs poorly for low-volume peritoneal disease. In a classic evaluation against surgical findings, the sensitivity of computed tomography fell to 28% for nodules less than 5 mm in thickness [6], and subsequent series have confirmed systematic underestimation of disease burden, particularly in the pelvis and small bowel mesentery [7,8]. Diffusion-weighted magnetic resonance imaging and artificial-intelligence-based approaches may improve detection but remain under evaluation [9,10]. A patient may therefore be staged as having localised, resectable disease, undergo an operation intended to be curative, and recur within the peritoneal cavity months later—not because the disease progressed, but because it was present and unseen from the outset.

Peritoneal lavage with cytological examination addresses precisely this blind spot. The technique requires no specialised equipment, adds only minutes to an operation already underway, and detects free tumour cells in patients whose peritoneum appears normal. This entity—positive cytology without visible peritoneal disease, designated CY1/P0—occupies a curious position in contemporary practice. In gastric cancer, CY1 has been classified as metastatic (M1) disease since its incorporation into the Japanese Gastric Cancer Association classification and subsequently the AJCC/UICC system [11,12,13,14], and staging laparoscopy with lavage is embedded in current international guidelines for locally advanced tumours [15,16,17]. A meta-analysis of comparative studies found positive cytology associated with substantially reduced overall survival (HR 3.46, 95% CI 2.77–4.31) [18], a finding consistent across large institutional series [19,20,21,22,23,24,25]. In colorectal, pancreatic and biliary cancer, the identical finding carries no formal staging status and, in most centres, is never sought.

This asymmetry is difficult to justify on biological grounds. The peritoneum is a common site of recurrence across all these tumour types, and the mechanism of dissemination—exfoliation of malignant cells from a serosa-breaching primary—does not obviously differ by organ of origin. Evidence outside the stomach is in fact reasonably substantial: a systematic review of colorectal series reported a weighted mean positivity of 13.2%, with positive lavage associated with worse survival in ten of eighteen studies and with increased recurrence in twelve [26], and meta-analysis has addressed its role in colorectal staging specifically [27]. Large institutional and multi-institutional series have identified lavage positivity as an independent predictor of peritoneal recurrence and of survival [28,29,30,31,32,33,34,35]. In resectable pancreatic cancer, meta-analysis links positive cytology to serosal invasion, lymphovascular invasion and markedly worse outcomes [36,37]. The evidence is not uniform; however, the EVOCAPE 2 multicentre prospective study found no prognostic significance for conventional peritoneal cytology in colorectal and gastric cancers [38], and comparative work has suggested that the biological behaviour of free cells may differ between colonic and gastric primaries [39]. Much of this discordance is attributable to methodological heterogeneity, with reported positivity ranging from 2.1% to 52% across studies depending on harvesting volume, timing and processing [26,40,41].

Interest in identifying these patients has nonetheless waned, in part because of negative trials of intraperitoneal chemotherapy. Adjuvant oxaliplatin HIPEC did not improve peritoneal metastasis-free survival in COLOPEC and conferred no survival benefit at five years [1,42]; second-look surgery with HIPEC did not improve disease-free survival in PROPHYLOCHIP-PRODIGE 15 [43]. These trials, however, evaluated a therapeutic strategy in patients selected by tumour stage, not a diagnostic test. A negative trial of prophylactic HIPEC does not establish that identifying occult dissemination is without value, and conflating the two risks discarding useful staging information because one proposed downstream use proved ineffective [5,44].

Contemporary abdominal surgery is increasingly framed in terms of precision—higher-resolution imaging, intraoperative navigation, robotic platforms. Peritoneal lavage is a reminder that precision also depends on asking the right question by simple means. We report a single-centre experience with peritoneal lavage cytology performed in consecutive patients undergoing surgery for advanced digestive malignancy in whom carcinomatosis had been excluded both radiologically and at operation. Our aims were to describe a standardised harvesting and cell block protocol; to determine the prevalence of occult positive cytology across tumour sites; to relate cytological status to the development of radiologically evident peritoneal carcinomatosis within 12 months; and to test whether cytological status could be predicted from the clinical and pathological characteristics of the primary tumour.

## 2. Materials and Methods

### 2.1. Study Design and Setting

This was a retrospective observational study conducted at the Department of Surgery, “Prof. Dr. O. Fodor”, Regional Institute of Gastroenterology and Hepatology, Cluj-Napoca, Romania, in collaboration with the Institute’s Department of Pathology. Cytological processing was performed using the department’s certified research infrastructure (ERIS identifier ERIO-2000-000K-0194). The study was approved by the Ethics Committee of the Regional Institute of Gastroenterology and Hepatology “Prof. Dr. O. Fodor”, Cluj-Napoca, and by the Ethics Committee of the University of Medicine and Pharmacy “Iuliu Hațieganu”, Cluj-Napoca (approval numbers and dates in the Institutional Review Board Statement). Reporting follows the STROBE statement for observational studies [45].

### 2.2. Patients

All patients undergoing peritoneal lavage during abdominal surgery for malignancy between January 2019 and December 2022 were identified from the departmental database. Lavage was performed in consecutive patients operated on by a single surgical team who met pre-defined criteria: a clinically staged T3 or T4 primary tumour, and no evidence of peritoneal carcinomatosis on preoperative cross-sectional imaging. During the study period, the operating team performed 45 procedures for clinically staged T3–T4 digestive malignancy without radiological peritoneal carcinomatosis, and peritoneal lavage was performed in all 45. Recruitment was therefore complete, and no eligible patient was omitted. Clinical staging determined eligibility because the decision to perform lavage was necessarily taken before operation; the pathological stage reported in Table 1 was established subsequently and did not influence selection.

Peritoneal status was classified as P0 only when carcinomatosis was absent on both preoperative cross-sectional imaging and systematic intraoperative inspection of the peritoneal cavity. Patients in whom carcinomatosis was identified by either assessment were classified as P1. This dual criterion is stricter than the intraoperative inspection alone used in most published series, and it means that patients classified as P0 here had passed two independent negative assessments before lavage was performed.

The primary analysis cohort comprised P0 patients with a digestive primary tumour. Patients with gynaecological or primary peritoneal malignancy were excluded from the primary analysis because of differing dissemination biology and are reported separately. P1 patients were not included in the primary analysis and are described separately in Section 3.1; lavage and cell block processing in these patients were identical to those used in the primary cohort.

### 2.3. Peritoneal Lavage Technique

The technique was uniform throughout the study period. Immediately on entering the abdomen, and before any tumour manipulation or mobilisation and before resection, 150–200 mL of 0.9% sodium chloride solution (B. Braun, Timișoara, Romania) was instilled into the peritoneal cavity in the region of the primary tumour. The fluid was gently agitated to disperse it and, after an interval of 10–15 s, a combined sample of approximately 10 mL was aspirated from the peritumoral region and from the pelvis. Sampling both sites was deliberate: peritumoral fluid captures cells exfoliated locally from the primary, while the pelvis is the site at which free intraperitoneal fluid pools by gravity, so the combined specimen samples both compartments. The specimen was transferred immediately to the Department of Pathology in a labelled sterile container.

Performing lavage before mobilisation is essential to the interpretation of the result: manipulation of a serosa-invading tumour can itself exfoliate cells into the cavity, and a specimen taken after resection cannot distinguish pre-existing dissemination from iatrogenic spillage. Timing relative to resection has been shown to influence positivity rates and is a recognised source of heterogeneity between published series [40,41]. Instillation was peritumoral rather than multi-quadrant, which differs from the whole-cavity lavage used in several gastric series; the protocol is reported here in full so that positivity rates can be interpreted against the method that produced them.

### 2.4. Cell Block Preparation

Specimens were processed according to the internal protocol of the Department of Pathology. Fluid was identified against patient records and centrifuged at 4000–5000 rpm for 5 min (DLAB DM0412, Thermo Scientific, Waltham, MA, USA). The supernatant was discarded and the pellet covered with 2 mL of 10% buffered dilute formaldehyde (Bio-Optica, Milano, Italy) for 10 min, then centrifuged again at 4000–5000 rpm for 5 min. After removal of the supernatant, the pellet was covered with approximately 1 mL of HistoGel (Bio-Optica, Milano, Italy) together with 2 mL of absolute ethanol (Bio-Optica, Milano, Italy), gently homogenised, and left at room temperature for 10 min. The resulting suspension was centrifuged at 4000–5000 rpm for 5 min; the supernatant was discarded and the sediment extracted and spread on filter paper. The product was placed in a cassette and thereafter processed identically to histopathological tissue specimens on a short programme.

Cytological diagnosis was based on standard morphological criteria for malignancy in serous specimens: the identification of a second cell population distinct from the reactive mesothelial background, showing nuclear enlargement with an increased nuclear-to-cytoplasmic ratio, hyperchromasia with coarse chromatin, irregular nuclear contours and prominent nucleoli. Architectural features preserved by cell block preparation—three-dimensional clusters, acinar or papillary groupings—and, where present, cytoplasmic mucin vacuolation or signet-ring morphology were taken as supporting evidence. Specimens were reported as positive (CY1) when these features were unequivocal and negative (CY0) otherwise. All specimens were reported by a single pathologist who received the specimen labelled only with a patient identifier and the specimen type, without clinical or operative information; independent second review was not performed. No specimen in the digestive cohort was reported as atypical, suspicious or non-diagnostic. Two specimens from non-digestive primaries reported over the same period—one primary peritoneal and one ovarian—were initially equivocal and were classified as positive on repeat review, confirming that this category was available to the reporting pathologist and was applied where the morphology warranted it.

### 2.5. Variables

The following were extracted from clinical and pathology records: age, sex, tumour site, histological type, differentiation grade, clinical TN stage, pathological TNM including lymphovascular (L, V), perineural (Pn) and margin (R) status, receipt of neoadjuvant chemotherapy, and cytology result.

### 2.6. Outcome

The study was designed as a prognostic association study. The exposure was peritoneal cytological status at operation (CY1 versus CY0), and the outcome was radiologically evident peritoneal carcinomatosis within 12 months of surgery, confirmed by contrast-enhanced CT. A fixed 12-month horizon was chosen because the surveillance protocol described in Section 2.7 delivered uniform CT assessment at four time points during the first postoperative year in every patient, giving complete and comparable ascertainment across the cohort. Patients free of carcinomatosis on the 12-month CT were classified as not having reached the endpoint. All events observed in this cohort occurred within the 12-month window, and no patient was censored before the endpoint horizon.

### 2.7. Postoperative Surveillance and Follow-Up

Postoperative surveillance followed a uniform institutional protocol. All patients underwent clinical review and contrast-enhanced computed tomography-Somatom Perspective (Siemens, Munich, Germany) of the thorax, abdomen and pelvis at 3, 6, 9 and 12 months after surgery, and observation continued to 24 months. Peritoneal carcinomatosis was diagnosed on contrast-enhanced CT by the presence of one or more of the following established descriptors: discrete peritoneal nodules or infiltrative soft-tissue masses; nodular or irregular parietal peritoneal thickening with contrast enhancement; omental haziness, stranding, nodularity or omental caking; mesenteric stranding, nodularity or a stellate configuration; and ascites, particularly when loculated. No diagnosis rested on clinical suspicion alone. Vital status was recorded at the 12-month assessment; mortality was not systematically ascertained thereafter. Follow-up data were assembled from two sources. The surgical database recorded the occurrence of peritoneal carcinomatosis within the surveillance year, and the oncological follow-up records documented the scheduled examination at which each radiological finding was reported. For patients who reached the endpoint, the surveillance examination at which the diagnosis was first made was established by cross-referencing the two. Exact calendar dates of surgery and of individual examinations were not retrievable from either source; events are therefore located to the surveillance interval at which they were detected rather than to a precise date.

Every patient in the primary cohort completed the 12-month assessment, so ascertainment of the primary endpoint was complete, and no patient was lost to follow-up before the endpoint horizon. Beyond 12 months, 28 of the 30 patients who had not developed carcinomatosis were observed to 24 months; the remaining two were last assessed at 12 months.

### 2.8. Statistical Analysis

Categorical variables are presented as counts and percentages with exact (Clopper–Pearson) 95% confidence intervals and compared using Fisher’s exact test. Continuous variables are presented as medians with interquartile ranges and compared using the Mann–Whitney U test. Predictive performance is reported as proportions with exact confidence intervals and is presented as a measure of prognostic association rather than of diagnostic accuracy. Two-sided *p* < 0.05 was considered statistically significant.

The primary analysis compares the proportion of patients reaching the endpoint within 12 months between cytological groups. Effect estimates are reported as relative risks with 95% confidence intervals, together with absolute risk differences. Odds ratios are given for comparability with the published literature but, because the outcome is frequent in this cohort, they overstate the relative risk and should not be read as one. Odds ratios for the comparisons in Table 1 were obtained by conditional maximum likelihood with exact confidence intervals, which is appropriate where cell counts are sparse.

A time-to-event analysis was also performed. Because exact dates were not retrievable, each event was assigned to the surveillance examination at which it was detected (3, 6, 9 or 12 months), and the data are therefore interval-censored: the examination that first demonstrated carcinomatosis is known, but not the point within the preceding interval at which the disease became detectable. Patients free of carcinomatosis were censored at the 12-month assessment, which all completed. Peritoneal carcinomatosis-free survival was estimated by the Kaplan–Meier method with events assigned to the interval of detection, and groups were compared by the log-rank test with tied events handled by the Mantel–Haenszel variance. The log-rank test assumes that censoring is non-informative, which holds here because all censoring was administrative at the 12-month assessment; because events are grouped at scheduled examinations, its *p* value should be regarded as approximate.

The hazard ratio was estimated from a discrete-time proportional hazards model. The data were expanded to one record per patient for each surveillance interval at risk, and a binomial regression with a complementary log–log link was fitted by maximum likelihood, with a separate baseline term for each interval and cytological status as the only covariate. Because no event occurred in the 6–9-month interval, it was combined with the 9–12-month interval. Under this model the exponentiated coefficient estimates the hazard ratio of the underlying continuous-time process, assuming that hazards are proportional across intervals; it is the grouped-time counterpart of the Cox model and is appropriate where events are known only to have occurred within discrete intervals. The 95% confidence interval was obtained by profile likelihood, which is preferable to a Wald interval when events are few, and significance was assessed by likelihood-ratio test. The proportional hazards assumption was examined by adding an interaction between cytological status and interval. These estimates describe the interval of detection under a fixed surveillance schedule rather than the timing of biological events.

The pooled analysis across tumour sites is exploratory. Because the cohort combines primaries with differing propensity for peritoneal dissemination, three sensitivity analyses were performed: restriction to patients without distant metastases (M0), restriction to patients who underwent resection, and restriction to both. Multivariable adjustment was not attempted, as 15 events cannot support a stable model; the sensitivity analyses are therefore the means by which confounding is addressed. Statistical analyses were performed using Python version 3.12.3 (Python Software Foundation, Wilmington, DE, USA) with the pandas (version 3.0.2), NumPy (version 2.4.4) and SciPy (version 1.17.1) packages. The discrete-time proportional hazards model was fitted by maximum likelihood using the optimisation routines in SciPy, and figures were produced with Matplotlib (version 3.10.8).

### 2.9. Use of Artificial Intelligence

General Artificial Intelligence was used for the editing of the manuscript (language and grammar corrections).

## 3. Results

### 3.1. Overall Series

Sixty-two peritoneal lavages were performed between January 2019 and December 2022. Carcinomatosis was identified at operation in 10 patients; the remaining 52 (83.9%) were P0, with no peritoneal disease on either preoperative imaging or intraoperative inspection. Of these 52, four had ovarian, one endometrial and two primary peritoneal malignancies and were excluded from the primary analysis, leaving 45 patients with digestive malignancy (Figure 1).

All 45 patients in the primary cohort completed the protocol-mandated 12-month CT assessment, so ascertainment of the primary endpoint was complete, and no patient was lost to follow-up before the endpoint horizon. Because completion of that assessment necessarily implies survival to it, overall survival at 12 months was 100%. Among the 30 patients who did not reach the endpoint, the median duration of observation was 24 months (IQR 24–24; range 12–24): 28 were observed to 24 months, and two were last assessed at 12 months. Observation did not differ materially between cytological groups. All three event-free CY1 patients were observed to 24 months, and the 27 event-free CY0 patients had a median observation of 24 months (range 12–24); all 13 CY1 and all 32 CY0 patients completed the 12-month assessment. All 15 events occurred within the 12-month surveillance window. The surveillance examination at which each event was detected is reported in Section 3.5; exact calendar dates were not retrievable, so events are located to that examination rather than to a precise date. Carcinomatosis was identified at operation in 10 patients in whom preoperative cross-sectional imaging had shown no peritoneal disease, representing 16.1% (95% CI 8.0–27.7) of the 62 lavages performed; this provides a direct measure within the present series of the insensitivity of cross-sectional imaging for macroscopic peritoneal disease described in the Introduction. All 10 were CY1. The group comprised seven gastric, two ovarian and one sarcomatous primary, and lavage and cell block processing were identical to those used in the primary cohort. Because it is small, predominantly gastric and includes non-digestive primaries, it is reported as a descriptive observation and is not used to support inference about the sensitivity of the technique in low-volume disease. These 10 patients are separate from, and not included in, the 45-patient primary cohort.

### 3.2. Cohort Characteristics

Median age in the primary cohort was 66 years (IQR 62–70); 29 patients (64.4%) were female; six (13.3%) received neoadjuvant chemotherapy. Fourteen patients (31.1%, 95% CI 18.2–46.6) had distant metastases at the time of surgery (M1), predominantly hepatic; the remaining 31 were M0. All patients received systemic therapy after operation: adjuvant therapy in the 40 who underwent resection and palliative chemotherapy in the five who did not, so that receipt of systemic treatment was uniform across cytological groups and could not differentially influence the endpoint. Individual regimens and completion rates were not uniformly recorded and are therefore not reported. Resection was macroscopically and microscopically complete (R0) in all 40 resected patients. Characteristics by cytological status are shown in Table 1.

Five patients did not undergo resection because the disease proved too locally advanced at exploration; all five received palliative chemotherapy. Four had gastric, and one a pancreatic primary; three were M1, four were CY1, and all five developed radiologically evident carcinomatosis within 12 months.

### 3.3. Prevalence of Occult Positive Cytology

Positive cytology was identified in 13 of 45 patients (28.9%, 95% CI 16.4–44.3) in whom carcinomatosis had been excluded by both preoperative imaging and intraoperative inspection. Prevalence varied substantially by primary site (Table 2).

Within the hepato-pancreato-biliary group, both pancreatic cases were CY1 (2/2), as were two of five biliary cases. Within the colorectal group, positivity clustered in recto-sigmoid and distal tumours (recto-sigmoid 3/7, transverse colon 1/4, sigmoid 1/10), with no positive result among right colon (0/5) or left colon (0/4) cases. Given the small denominators, these site-level differences are descriptive and should not be over-interpreted.

Among the seven excluded gynaecological and primary peritoneal P0 cases—four ovarian, two primary peritoneal and one endometrial—all were CY1.

### 3.4. Cytological Status and Radiologically Evident Carcinomatosis Within 12 Months

Radiologically evident peritoneal carcinomatosis was diagnosed within 12 months in 15 of 45 patients (33.3%); every event occurred within the surveillance year, and no patient reached the endpoint thereafter. The association with cytological status was strong (Table 3). The endpoint was reached by 10 of 13 CY1 patients (76.9%, 95% CI 46.2–95.0) and 5 of 32 CY0 patients (15.6%, 95% CI 5.3–32.8), an absolute risk difference of 61.3 percentage points and a relative risk of 4.92 (95% CI 2.09–11.62; Fisher’s exact *p* = 0.00018). The corresponding odds ratio is 18.0 (95% CI 3.62–89.6); because the outcome is frequent in this cohort, the odds ratio substantially overstates the relative risk, and the latter is the more interpretable estimate.

Three sensitivity analyses were performed to address confounding by baseline disease extent. Restricted to the 31 patients without distant metastases, the endpoint was reached by 4 of 6 CY1 and 3 of 25 CY0 patients (66.7% versus 12.0%; RR 5.56, 95% CI 1.67–18.50; *p* = 0.014). Restricted to the 40 patients who underwent resection, it was reached by 6 of 9 CY1 and 4 of 31 CY0 patients (66.7% versus 12.9%; RR 5.17, 95% CI 1.85–14.39; *p* = 0.003). Restricted to the 29 patients who were both M0 and resected, it was reached by 3 of 5 CY1 and 2 of 24 CY0 patients (60.0% versus 8.3%; RR 7.20, 95% CI 1.59–32.52; *p* = 0.024). The association was therefore preserved, and the relative risk was larger rather than smaller, in each of the more restricted and more homogeneous subgroups.

Two potential confounders are reported here rather than deferred to the Discussion. Cytological positivity was more frequent in patients with distant metastatic disease (CY1 in 7 of 14 M1 patients versus 6 of 31 M0 patients, 50.0% versus 19.4%), although this difference did not reach statistical significance (*p* = 0.072), and distant metastatic disease was associated with reaching the endpoint (8 of 14 versus 7 of 31, 57.1% versus 22.6%, *p* = 0.039). Metastatic status therefore has the characteristics of a potential confounder. Restriction to M0 patients did not, however, reduce the relative risk (5.56 compared with 4.92 in the full cohort), so these data provide no evidence that the pooled estimate is inflated by distant metastatic disease, although the confidence intervals are too wide to exclude it. Tumour site behaves similarly: cytological positivity varied from 66.7% in gastric to 16.7% in colorectal primaries (Table 2), and site is plausibly associated with the outcome as well. With 15 events, multivariable adjustment for either variable was not feasible, and the pooled estimate should be read as exploratory.

Five patients with negative cytology nonetheless developed peritoneal carcinomatosis within 12 months. Conventional cytology detects free tumour cells only when they are present in the aspirated sample in sufficient number and are morphologically unambiguous; low-volume dissemination below this threshold, or cells confined to peritoneal surfaces rather than free in the cavity, will not be captured. This limitation is intrinsic to morphological cytology and is the principal rationale for the immunocytochemical and molecular adjuncts discussed below [26,46,47].

### 3.5. Timing of Detection and Peritoneal Carcinomatosis-Free Survival

The surveillance examination at which carcinomatosis was first identified was recoverable by cross-referencing the surgical database with the oncological follow-up records in all 15 patients who reached the endpoint. Eight events were detected at the 3-month examination, six at 6 months and one at 12 months; none was detected at the 9-month examination. The median interval of detection was the 3-month scan in CY1 patients and the 6-month scan in CY0 patients.

Detection occurred earlier and more frequently in cytology-positive patients. Six of 13 CY1 patients (46.2%) already had radiologically evident carcinomatosis at the first surveillance examination three months after surgery, compared with 2 of 32 CY0 patients (6.3%). By the 6-month examination, the corresponding figures were 10 of 13 (76.9%) and 4 of 32 (12.5%).

Estimated peritoneal carcinomatosis-free survival in CY1 patients was 53.8% at 3 months and 23.1% at 6 months, and remained at 23.1% at 12 months. In CY0 patients the corresponding estimates were 93.8%, 87.5% and 84.4% (Figure 2). The difference between groups was significant on log-rank testing (χ^2^ = 18.43, 1 df, *p* = 0.000018). In the discrete-time proportional hazards model, the hazard ratio for CY1 was 9.07 (95% profile-likelihood CI 3.15–29.72; likelihood-ratio χ^2^ = 16.46, 1 df, *p* = 0.00005). There was no evidence against proportional hazards (interaction *p* = 0.92), the interval-specific estimates being 9.6 for the first three months and 8.6 thereafter.

These estimates describe the interval at which disease was detected under a fixed surveillance schedule, not the point at which it became biologically established; because exact dates were not retrievable, the data are interval-censored, and the curves should be read as step functions defined by the scheduled examinations rather than as continuous estimates of risk.

### 3.6. Association Between Cytological Status and Tumour Characteristics

None of the clinical or pathological variables examined reached statistical significance in relation to cytological status (Table 1). pT4 category, nodal positivity, grade 3 differentiation, signet-ring or mucinous histology, receipt of neoadjuvant chemotherapy, age and sex were all non-significant, although nodal positivity was more frequent among CY1 patients (87.5% versus 59.3%) and the direction of this difference is consistent with published series. For pT4 category the point estimate ran counter to expectation, with CY1 less frequent among pT4 tumours (37.5% versus 59.3%). This comparison should be read with the selection criteria in mind: because lavage was restricted to clinically staged T3 and T4 tumours, the cohort spans a narrow range of local invasion, which limits the capacity to detect an association between depth of invasion and cytological status.

Because eligibility was determined by clinical stage while the analysis uses pathological stage, we examined their concordance. Both were evaluable in 35 patients and agreed in 30 (85.7%); four tumours were clinically overstaged and one understaged. Substituting clinical for pathological T category did not alter the association with cytological status (*p* = 0.73 using cT versus *p* = 0.42 using pT), and restricting the analysis to concordant patients likewise did not (*p* = 0.66). The inverse point estimate is therefore not attributable to staging discordance.

These comparisons involve limited numbers and wide confidence intervals, and the absence of statistical significance should not be read as evidence that no association exists; associations reported in larger series may well hold in this setting. What can be said is that, within this cohort, no single conventional feature identified the cytology-positive patients reliably enough to have served as a substitute for performing the lavage.

## 4. Discussion

In patients undergoing surgery for advanced digestive malignancy in whom peritoneal disease had been excluded twice over—once by preoperative cross-sectional imaging and again by direct intraoperative inspection—nearly one in three nonetheless harboured free intraperitoneal tumour cells. That finding was associated with carcinomatosis becoming radiologically evident within 12 months, with an odds ratio of 18.0 (95% CI 3.62–89.6) and a positive predictive value of 76.9%, and none of the clinicopathological characteristics of the primary tumour examined here identified it reliably enough to have served in its place. All patients with carcinomatosis were cytology-positive, confirming that the technique performs as expected when disease is unambiguously present.

The dual criterion for P0 status deserves emphasis, because it distinguishes this series from much of the published literature. Most studies define the absence of peritoneal disease by intraoperative inspection alone, and a smaller number by imaging alone. Requiring both raises the threshold for entry into the cohort: every patient counted here had already survived two independent negative assessments before the lavage was taken. The 28.9% positivity rate is therefore a residual after conventional staging has been exhausted, not a figure obtained in patients who were incompletely evaluated. It also means the rate is likely to be conservative relative to series using a single negative criterion, since patients with low-volume disease visible to one modality but not the other were classified as P1 and excluded from the primary cohort.

### 4.1. Non-Redundancy of the Information

The central observation is not that CY1 is associated with peritoneal recurrence—that is established in gastric cancer [18,19] and increasingly supported in colorectal [26,28,33,34] and pancreatic disease [36,37]. It is that CY1 was not reliably identified by anything else in the diagnostic pathway. Imaging did not identify these patients, by definition of the cohort. Direct inspection of the peritoneum did not identify them. And in our series, no pathological feature examined—depth of invasion, nodal burden, grade, or adverse histological subtype—distinguished them from cytology-negative patients with statistical significance, although the numbers involved are small.

This bears on how the test might eventually be used, a question the present study can inform but not settle. If CY1 tracked closely with T4 status or signet-ring histology, selective lavage in high-risk cases would capture most positives and routine lavage would be difficult to justify. In our cohort, positives were distributed across the range of stage and histology represented, so a risk-stratified approach based on these features alone would have missed a proportion of them. Whether the same holds across a wider range of stage remains to be established. The test is inexpensive, adds minutes to an operation already underway and carries no identified morbidity, and it supplies information that was not obtainable by other means in this cohort. Whether that justifies routine adoption is a separate question, which a retrospective single-centre series of this size cannot answer; the evidence that would be required is set out in Section 4.7.

This null finding must nonetheless be stated with care, because it is not the consensus position. A recent synthesis of lavage technique across tumour entities reports that serosal invasion is associated with higher detection rates of free tumour cells than non-serosa-invading tumours [41], and pooled analyses in pancreatic and colorectal cancer have linked positive cytology to T stage, nodal status and lymphovascular invasion [26,36]. Our cohort did not reproduce these associations at conventional levels of significance, and with 13 cytology-positive patients it was not designed to do so; the confidence intervals around each comparison are wide enough to accommodate clinically meaningful effects. We regard these published associations as likely to be real, and our findings should not be read as contradicting them. The restriction of the cohort to clinically staged T3–T4 disease further narrows the range of local invasion across which such an association could be observed. The appropriate conclusion is narrower than a claim of true independence: within this series, no conventional feature was a usable surrogate for the test, and clinicians selecting patients for lavage on the basis of T category alone would have missed positive results. Whether stage-based selection is adequate in larger populations remains an open question that this study cannot settle.

### 4.2. Occult Baseline Disease Versus Subsequent Progression

The endpoint of this study requires careful interpretation. Free intraperitoneal tumour cells identified at the time of operation most plausibly represent peritoneal dissemination that was already present, but below the detection threshold of both cross-sectional imaging and direct intraoperative inspection. On that reading the surveillance period records not the development of new metastatic disease, but the interval over which pre-existing microscopic disease became radiologically apparent.

This distinction matters for how the test should be understood. On this reading lavage cytology functions as a staging investigation, describing disease extent at the time of surgery, rather than as a marker predicting future biological behaviour; we note, however, that the present study cannot establish which interpretation is correct. Such a staging role is the one the finding already occupies in gastric cancer, where CY1 defines M1 disease rather than an elevated risk of later recurrence [11,12,13,14]. Outside the stomach CY1 carries no formal M1 designation, and we do not propose that the present data establish one.

The two mechanisms are not mutually exclusive, and our data cannot separate them for any individual patient. Positive cytology confirms the presence of free malignant cells at operation, but the study cannot establish whether the macroscopic disease that followed arose from those cells, from peritoneal surface implants already present but undetected at inspection, or from dissemination occurring subsequently. All events in this cohort occurred within the first postoperative year, an interval more readily explained by the growth of disease already established at operation than by de novo dissemination followed by progression to radiological visibility; but this is an inference from timing rather than a demonstration. The distinction bears directly on how the proportions in Table 4 should be read: they describe the relation between cytological status and disease becoming radiologically evident within a defined observation window, not how well lavage forecasts peritoneal failure across the whole natural history of the disease. A negative lavage excludes detectable free tumour cells on the day of surgery; it does not exclude subsequent dissemination, and the five cytology-negative patients who developed carcinomatosis may represent either disease below the detection threshold of the technique or genuine later spread.

A further consideration concerns the endpoint itself. Carcinomatosis was diagnosed radiologically, and no case was confirmed histologically, so misclassification cannot be formally excluded: postoperative change, reactive inflammation and tuberculous peritonitis can each reproduce the appearances listed in Section 2.7. Two points bear on the likely magnitude. All diagnoses were made on protocol-mandated surveillance imaging in patients under oncological follow-up for advanced disease, a setting in which the prior probability of peritoneal metastasis is high and these mimics uncommon; and non-differential misclassification would bias the observed association towards the null rather than away from it. Differential misclassification is the more relevant concern, since cytological results were known to the treating team and surveillance imaging was reported within the same institution; this is addressed in the Limitations.

### 4.3. The Gastric–Non-Gastric Asymmetry

Peritoneal lavage occupies radically different positions across digestive tumour types. In gastric cancer CY1 alone defines M1 disease and is embedded in AJCC/UICC and JGCA staging [11,12,13,14] and in ESMO and NCCN guidance [15,16]. Elsewhere in the digestive tract the identical finding is not staged and is rarely sought. Our gastric positivity rate (66.7%) is higher than typically reported, almost certainly reflecting selection of locally advanced cases in a very small subgroup, and should not be taken as a population estimate.

The more interesting observation concerns the non-gastric groups. Half of our hepato-pancreato-biliary patients and one in six colorectal patients were CY1 despite a normal-appearing peritoneum, and these patients recurred peritoneally. Our colorectal rate sits close to the weighted mean of 13.2% reported in systematic review [26] and above rates from series using conventional cytology alone [29,30], which is consistent with the hypothesis that cell block preparation affords better cellular yield than direct smears. We emphasise that this is a hypothesis derived from the external literature on serous effusion cytology [46,48] and not a conclusion supported by the present data: no paired comparison of smear and cell block preparations was performed, and our study cannot test it. Such a comparison is identified below as a requirement for prospective evaluation.

It would be incomplete to present this literature as uniformly supportive. The EVOCAPE 2 multicentre prospective study concluded that conventional peritoneal cytology lacked prognostic significance in both colorectal and gastric cancers [38], and earlier comparative work suggested that microscopic peritoneal dissemination may carry different weight in colonic than in gastric disease [39]. These findings deserve direct engagement rather than omission. Two considerations bear on them. First, conventional smear cytology and cell block preparation are not the same test: cell block methods improve cellular yield and diagnostic accuracy in serous effusions, and permit immunocytochemical adjudication of morphologically ambiguous cases [46,48,49]. Studies reporting null results using smear-based methods do not necessarily generalise to cell block-based practice. Second, the extraordinary range of reported positivity in colorectal cancer—from 2.1% to 52% [26]—is itself evidence that these studies are not measuring the same thing. Harvesting volume, irrigated regions, timing relative to mobilisation, and processing method all vary, and all affect yield [40,41]. Rather than undermining the case for lavage, this heterogeneity is the strongest available argument for protocol standardisation, which is why we report our technique in reproducible detail.

### 4.4. Relation to the Intraperitoneal Chemotherapy Trials

Enthusiasm for identifying occult peritoneal disease in colorectal cancer diminished after COLOPEC and PROPHYLOCHIP-PRODIGE 15 reported no benefit from adjuvant or second-look HIPEC [1,43], with COLOPEC confirming the absence of a survival difference at five years [42]. The subsequent HIPECT4 trial, which examined concurrent HIPEC in locally advanced colorectal cancer, reported improved locoregional control [50], and an individual patient data meta-analysis has since examined these trials together [51]; the field is not settled, but prophylactic HIPEC is not currently standard [44].

It is important to be explicit: our data do not support prophylactic HIPEC, and we do not propose it. The trials cited tested a therapeutic intervention in patients selected by tumour stage—T4 or perforated disease—not by cytological status. The value of a diagnostic test is a separate question from the efficacy of one downstream treatment [5,44]. Notably, the one study that did select patients on the basis of positive lavage cytology for intraperitoneal chemotherapy reported reduced peritoneal recurrence [52], and the COLOPEC 2 design incorporates repeat laparoscopy for early detection in high-risk patients [53]—an acknowledgement that identification, not only treatment, is part of the problem. Whether identifying CY1 patients should influence surveillance or systemic therapy is a question the present data cannot answer; the finding bears more directly on the design of future trials, which have, to date, largely enrolled on the basis of T category rather than demonstrated peritoneal dissemination. A trial population defined by CY1 would be materially enriched for the outcome such trials aim to prevent.

### 4.5. Cohort Heterogeneity

The primary cohort combines gastric, pancreatic, biliary, colorectal and appendiceal primaries, and the rationale for pooling them requires statement. The research question concerns the diagnostic yield of a surgical technique rather than the natural history of any one tumour. The mechanism under study—exfoliation of malignant cells from a serosa-breaching primary into the peritoneal cavity, and their recovery by saline lavage—is common to all these sites, and the harvesting and processing protocol was identical throughout. Pooling was therefore appropriate for the primary question of whether the technique yields information not otherwise available.

The composition of the cohort shapes what the pooled figure means. These tumours differ in their propensity for peritoneal dissemination, and our site-specific rates track those differences closely—66.7% in gastric, 50.0% in hepato-pancreato-biliary and 16.7% in colorectal primaries, a gradient consistent with the published literature and itself a form of internal coherence. The overall figure of 28.9% is therefore a composite determined by case mix rather than a rate transferable to any single tumour type, which is why positivity is reported by site in Table 2.

Inference is correspondingly restricted. The colorectal group (*n* = 30) is the only one large enough to support conclusions in its own right; the gastric, hepato-pancreato-biliary and appendiceal groups are reported descriptively. The association between cytological status and carcinomatosis was not attributable to any single site, since events occurred throughout the cohort, but its magnitude within a given tumour type cannot be estimated from these data and will require site-specific series.

### 4.6. External Validity

Consecutive enrolment under pre-defined criteria, a single operating team and one processing protocol give this series a degree of internal consistency uncommon in retrospective work on peritoneal cytology, where variation in technique is the principal source of discordance between published rates [26,40,41]. That consistency is bought at some cost to breadth. Eligibility required clinically staged T3 or T4 disease, so the findings do not extend to earlier-stage tumours; the operating team practises at a tertiary centre with a substantial hepato-pancreato-biliary caseload, which shapes case mix and the thoroughness of intraoperative assessment; and cytological interpretation rests on a single department applying one cell block protocol.

The direction of any resulting bias can be stated with reasonable confidence. Because P0 status required negative findings on both preoperative imaging and systematic intraoperative inspection, this cohort is likely depleted of patients with low-volume visible disease, so the observed rate of 28.9% is more plausibly conservative than inflated. Multicentre validation with standardised harvesting and independent cytological review is the natural next step, and the protocol reported here is offered as a candidate standard for it.

### 4.7. Clinical Implications

The association reported here is prognostic, and the questions it raises for practice are hypotheses for prospective testing rather than recommendations. This exploratory study was not designed to evaluate, and does not support, routine peritoneal lavage cytology or any change to postoperative surveillance or treatment.

Three such hypotheses follow from the data. First, because 6 of 13 CY1 patients (46.2%) already had radiologically evident carcinomatosis at the 3-month examination, compared with 2 of 32 CY0 patients (6.3%), it may be asked whether earlier or more frequent imaging in cytology-positive patients would alter the course of disease; the present data show only when disease was detected under a fixed schedule, and cannot show that earlier detection would be of benefit. Second, it may be asked whether cytological status should inform multidisciplinary discussion of systemic therapy; no trial has shown that modifying systemic treatment on the basis of cytology alters outcome outside gastric cancer. Third, trials of intraperitoneal therapy have to date enrolled patients on the basis of T category and therefore include many who would never have developed peritoneal disease; selection on cytological status might enrich such trials for the outcome they seek to prevent.

Before any of these could inform practice, prospective multicentre evaluation would need to show that the finding is reproducible, that it is independent of established prognostic factors, that acting on it changes management in a defined way, that the change is affordable, and that it confers a patient-relevant benefit in survival or in freedom from symptomatic peritoneal disease. Until then the appropriate claim is limited to the association itself: that in this exploratory series cytological status identified a group at substantially higher near-term risk of radiologically evident peritoneal disease.

### 4.8. Future Perspectives

Several directions follow from this work. Prospective, consecutive, multicentre evaluation is needed to establish site-specific positivity rates free of selection bias, and the design requirements can be specified from the limitations of the present series. Exact dates of surgery and of each surveillance examination should be recorded, so that peritoneal carcinomatosis-free survival can be analysed as a continuous time-to-event outcome rather than by assignment to surveillance intervals as was necessary here. Mortality should be ascertained systematically beyond the primary endpoint, so that overall and disease-free survival can be reported. Cytological specimens should undergo independent second review with a formal measure of inter-observer agreement, and surveillance imaging should be reported by radiologists blinded to cytological status, since neither was possible in this retrospective series. A paired comparison of direct smear and cell block preparations from the same specimen would test the hypothesis, advanced above on the basis of the external literature, that cell block methods afford superior cellular yield. Molecular characterisation, including mismatch repair and RAS/BRAF status, should be collected systematically, as these markers are relevant to peritoneal dissemination in colorectal cancer and were too incompletely recorded here to permit analysis. Harvesting and processing protocols require standardisation, given the methodological heterogeneity described above [26,40,41]. Sensitivity may be improved by immunocytochemistry, which yields higher detection rates than conventional cytology alone; panels based on Ber-EP4, MOC-31 and calretinin are well validated for distinguishing adenocarcinoma from reactive mesothelium in serous specimens [46,49], and claudin-4 has recently been proposed as a single discriminating marker [49]. Peritoneal tumour DNA is an emerging candidate biomarker for occult peritoneal disease with promising prognostic performance [47]. Intraoperative adjuncts such as indocyanine green fluorescence [54] and artificial intelligence applied to cross-sectional imaging [10] are being investigated for detection of peritoneal metastasis, and the relationship between these approaches and cytological confirmation deserves study—they are complementary rather than competing. Finally, the question of whether CY1 should carry formal staging weight outside the stomach remains open and will require prospectively collected, adequately powered data.

### 4.9. Limitations

Several limitations qualify these findings beyond the questions of heterogeneity and generalisability considered above. The study is retrospective and single-centre. Cytological results were available to the treating team and surveillance imaging was reported within the same institution, so outcome ascertainment was not blinded to exposure status; an influence of a known positive result on the reading of equivocal peritoneal findings cannot be excluded. Cytological interpretation was performed by a single pathologist without independent second review, and because the threshold for reporting unequivocal malignancy is operator-dependent, the observed positivity rate should be read with that in mind.

Further constraints apply to the data themselves. The number of events precluded multivariable adjustment, so the reported associations are unadjusted, and the study cannot establish that cytological status is an independent prognostic factor; the number of patients limits power for subgroup comparison, although the cohort is in keeping with published single-centre series of peritoneal lavage cytology outside gastric cancer. Pathological T and N categories were unavailable in ten patients: five did not undergo resection, and staging was incomplete in a further five who did. Exact calendar dates of surgery and of individual surveillance examinations were not retrievable, so the time-to-event analysis presented in Section 3.5 is interval-censored: the examination at which each event was detected is known, but the point within the preceding interval at which the disease became detectable is not. The survival estimates therefore describe detection under a fixed surveillance schedule rather than the underlying timing of biological events; the median interval of detection can be given only to the resolution of that schedule, and the hazard ratio rests on the assumption of proportional hazards across intervals. All 45 patients were alive at the 12-month assessment, which every patient completed. Mortality was not systematically ascertained beyond that point, and patients subsequently managed at other institutions were not traced; overall and disease-free survival were therefore not analysed and cannot be inferred from these data. Survival is a clinically important outcome in this population, and its systematic collection should form part of any prospective evaluation. Molecular characterisation was not uniformly available: mismatch repair and RAS/BRAF status were determined in some but not all colorectal patients during the study period, and the data were too incomplete to permit analysis. Because these markers are relevant to the biology of peritoneal dissemination in colorectal cancer, their absence is a limitation of the present series.

## 5. Conclusions

In this exploratory single-centre retrospective series of patients undergoing surgery for advanced digestive malignancy, in whom peritoneal carcinomatosis had been excluded by both preoperative imaging and intraoperative inspection, peritoneal lavage cytology identified free intraperitoneal tumour cells in 28.9%. Radiologically evident carcinomatosis developed within 12 months in 76.9% of cytology-positive and 15.6% of cytology-negative patients (relative risk 4.92, 95% CI 2.09–11.62), and was detected earlier in cytology-positive patients (hazard ratio 9.07, 95% CI 3.15–29.72). These are unadjusted associations in a small and heterogeneous cohort, and they do not establish that cytological status is an independent prognostic factor. Nor do they support routine peritoneal lavage cytology or any change to postoperative surveillance; whether either is justified is a question for adequately powered prospective studies, for which this series provides a rationale and a reproducible protocol.

## Figures and Tables

**Figure 1:**
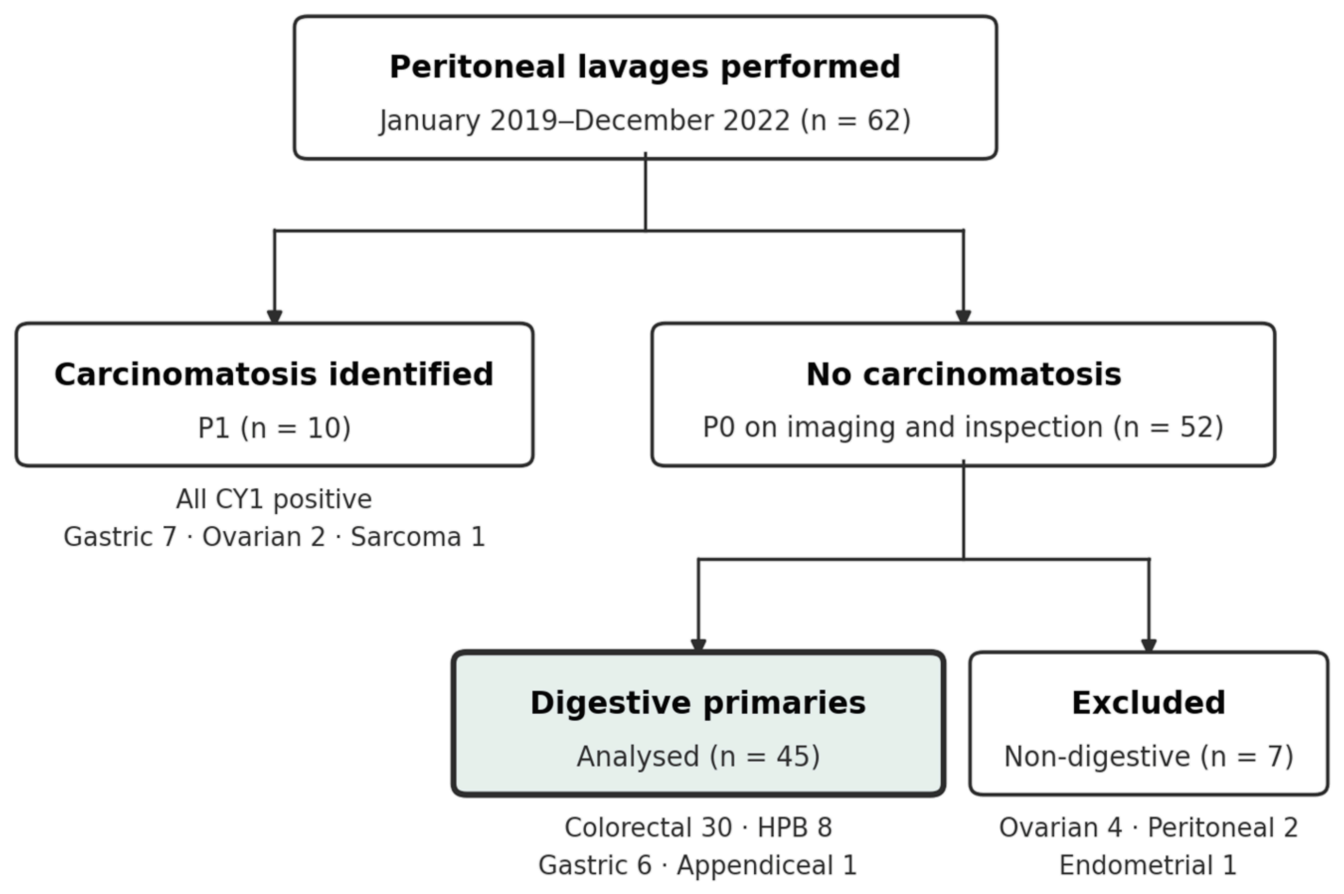
Study flow diagram. Sixty-two lavages were performed; 10 patients had carcinomatosis (P1) and 52 were P0 on both imaging and intraoperative inspection. Seven non-digestive primaries were excluded, leaving 45 patients in the primary analysis cohort.

**Figure 2:**
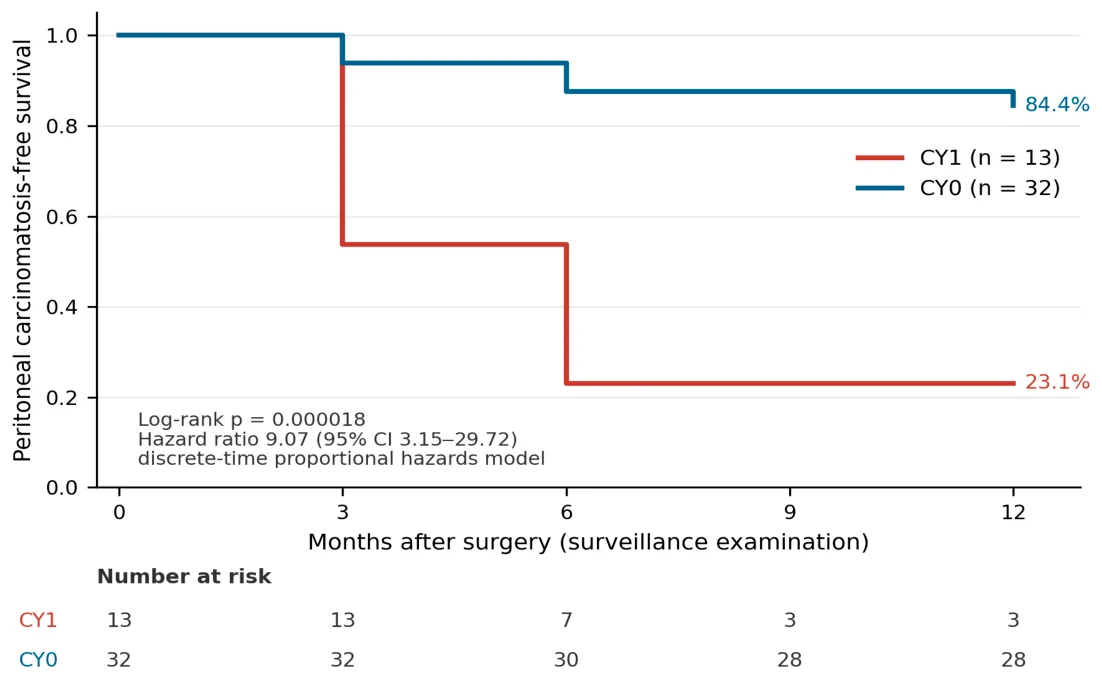
Peritoneal carcinomatosis-free survival by cytological status, with events assigned to the surveillance interval at which they were detected. Patients free of carcinomatosis were censored at the 12-month assessment, which all 45 completed. Log-rank *p* = 0.000018; hazard ratio from the discrete-time proportional hazards model 9.07 (95% profile-likelihood CI 3.15–29.72).

**Table 1:** Characteristics of the primary cohort (digestive malignancy, P0 on both imaging and intraoperative inspection, *n* = 45) by peritoneal cytology status. Event counts and denominators are given for every row. Odds ratios were obtained by conditional maximum likelihood with exact 95% confidence intervals.

Variable	CY1 (*n* = 13)	CY0 (*n* = 32)	Odds Ratio (95% CI)	*p*
Age, years, median (IQR)	63 (60–71)	67 (63–70)	—	0.41
Gender, female	10/13 (76.9%)	19/32 (59.4%)	2.24 (0.45–15.1)	0.32
M1 stage at surgery	7/13 (53.8%)	7/32 (21.9%)	4.02 (0.85–20.4)	0.07
pT4 category	3/8 (37.5%)	16/27 (59.3%)	0.42 (0.05–2.7)	0.42
Node positive (pN)	7/8 (87.5%)	16/27 (59.3%)	4.63 (0.48–236.3)	0.22
Grade 3	0/9 (0%)	4/27 (14.8%)	0.00 (0.00–4.7)	0.55
Signet-ring or mucinous	4/13 (30.8%)	7/32 (21.9%)	1.57 (0.27–8.1)	0.70
Neoadjuvant chemotherapy	2/13 (15.4%)	4/32 (12.5%)	1.27 (0.10–10.4)	1.00

T and N categories are pathological and were evaluable in 35 patients: five did not undergo resection, so no pathological stage was obtainable, and pathological data were incomplete in a further five. Differentiation grade was evaluable in 36 patients. Denominators are stated in each cell. Wide confidence intervals reflect sparse cell counts and should not be interpreted as evidence of absence of association.

**Table 2:** Occult positive peritoneal cytology by tumour site (*n* = 45).

Site	*n*	CY1	Rate (95% CI)
Gastric	6	4	66.7% (22.3–95.7)
Hepato-pancreato-biliary	8	4	50.0% (15.7–84.3)
Colorectal	30	5	16.7% (5.6–34.7)
Appendiceal	1	0	—
Total	45	13	28.9% (16.4–44.3)

**Table 3:** Peritoneal cytology and radiologically evident carcinomatosis within 12 months (*n* = 45).

	Carcinomatosis	No Carcinomatosis	Total
CY1	10	3	13
CY0	5	27	32
Total	15	30	45

Relative risk 4.92 (95% CI 2.09–11.62); absolute risk difference 61.3 percentage points; odds ratio 18.0 (95% CI 3.62–89.6); Fisher’s exact *p* = 0.00018.

**Table 4:** Predictive performance of peritoneal cytological status for radiologically evident peritoneal carcinomatosis within 12 months (*n* = 45). These are measures of prognostic association, not of diagnostic accuracy against a reference standard for disease present at operation; the last two rows correspond to what would conventionally be termed sensitivity and specificity but are expressed as proportions to avoid that implication. All proportions depend on the frequency of the endpoint in this cohort (33.3%) and would differ in populations with a different underlying risk.

Measure	Value (95% CI)
Proportion of CY1 patients reaching the endpoint	76.9% (46.2–95.0)
Proportion of CY0 patients reaching the endpoint	15.6% (5.3–32.8)
Relative risk	4.92 (2.09–11.62)
Absolute risk difference	61.3 percentage points
Odds ratio	18.0 (3.62–89.6)
Proportion of endpoint events preceded by CY1	66.7% (38.4–88.2)
Proportion of endpoint-free patients who were CY0	90.0% (73.5–97.9)

## Data Availability

The data presented in this study are available on request from the corresponding author. The data are not publicly available due to privacy restrictions.

## Abbreviations

The following abbreviations are used in this manuscript:

CI	Confidence interval
CY0	Negative peritoneal lavage cytology
CY1	Positive peritoneal lavage cytology
HIPEC	Hyperthermic intraperitoneal chemotherapy
HR	Hazard ratio
IQR	Interquartile range
P0	No peritoneal carcinomatosis on imaging or at operation
P1	Macroscopic peritoneal carcinomatosis present
RR	Relative risk
STROBE	Strengthening the Reporting of Observational Studies in Epidemiology
